# Sulfatase modifying factor 2 as a predictive biomarker for urothelial carcinoma

**DOI:** 10.1007/s12672-025-01859-y

**Published:** 2025-02-07

**Authors:** Wei-Ting Kuo, Yi-Chen Lee, Jia-Bin Liao, Ching-Jiunn Tseng, Yi-Fang Yang

**Affiliations:** 1 Division of Urology, Department of Surgery, Kaohsiung Veterans General Hospital, Kaohsiung, Taiwan; 2 Institute of Clinical Medicine, National Yang Ming Chiao Tung University, Taipei, Taiwan; 3 Department of Anatomy, School of Medicine, College of Medicine, Kaohsiung Medical University, Kaohsiung, Taiwan; 4 Department of Pathology and Laboratory Medicine, Kaohsiung Veterans General Hospital, Kaohsiung, Taiwan; 5 Department of Medical Education and Research, Kaohsiung Veterans General Hospital, No. 386, Dajhong 1st Rd., Zuoying Dist, Kaohsiung, 813414 Taiwan

**Keywords:** Sulfatases, SUMF2, FBXW7, Urothelial carcinoma

## Abstract

Sulfatases mediate the sulfation level of cell-surface heparan sulfates to regulate signal transduction, thereby promoting cancer progression. Sulfatase activation requires a sulfatase modifying factor (SUMF) for the modification of its catalytic domain. The role of the SUMF family in urothelial carcinoma (UC) has not been adequately evaluated. In this study, we used an online database and immunohistochemistry to assess genetic changes and the mRNA and protein expression of SUMFs and related candidate targets in UC. We found that *SUMF1* and *SUMF2* were amplified in UC tissues. High *SUMF2* mRNA levels were associated with poor overall survival (OS) and disease-free survival (DFS) in bladder UC (BLCA) from The Cancer Genome Atlas (TCGA) dataset. High SUMF2 protein levels were associated with grade (*P* < 0.001), T status (*P* = 0.01), and stage (*P* = 0.006) in patients with BLCA. We also examined SUMF2 expression levels in upper tract UC (UTUC). SUMF2 expression was associated with stage (*P* = 0.046), poor OS (*P* = 0.0022), and DFS (*P* = 0.019) in patients with UTUC. Knockdown of *SUMF2* significantly reduced the migration and invasion abilities of 5637 cells. Furthermore, *SUMF2* mRNA levels negatively correlated with *FBXW7* mRNA levels in BLCA. The *SUMF2*^high^/*FBXW7*^low^ expression profile predicted the worst survival in BLCA. Taken together, SUMF2 expression is linked to unfavorable clinical outcomes in patients with UC and may serve as a useful prognostic biomarker for UC staging.

**Supplementary Information:**

The online version contains supplementary material available at 10.1007/s12672-025-01859-y.

## Introduction

Urothelial carcinoma (UC) may develop along the epithelial lining of any segment of the urinary tract, from the bladder to the renal calyces. A specific subset, upper tract UC (UTUC), manifests as tumors in the renal pelvis or ureter, with ureteral tumors representing approximately one-third of all UTUC cases. While UTUC is a relatively rare disease globally [1], its prevalence in Taiwan can be notably high, constituting up to 30% of all UC cases. The elevated incidence of this form of cancer in Taiwan has been associated with internal factors, such as genetic susceptibility, and external environmental or occupational influences, such as exposure to groundwater arsenic and the widespread use of Chinese herbal medicine [2].

Owing to its frequent occurrence in the urinary bladder, the diagnosis and management of UC typically centers around this specific anatomical location. However, clinicians should remain vigilant of the potential involvement of other segments of the urinary tract [1]. A comprehensive approach involves a nuanced understanding of the pathological characteristics, staging, and treatment options associated with UC [3, 4]. By recognizing the diverse manifestations and potential spread of UC beyond the bladder, clinicians can tailor diagnostic and therapeutic strategies accordingly. This includes consideration of UTUC, which necessitates distinct deliberations in terms of staging and treatment modalities [5]. To provide appropriate care for patients grappling with this urological malignancy, a thorough understanding of the intricacies of UC is essential. This ensures that clinicians can deliver comprehensive and effective management strategies, address the unique aspects of each case, and optimize outcomes for those affected by this challenging condition.

Sulfatase modifying factor (SUMF), a member of the formylglycine-generating enzyme (FGE) family, is located in the endoplasmic reticulum and is responsible for the post-translational formation of Cα-formylglycine (FGly). It catalyzes the oxidation of cysteine in the active site of sulfatases (SULFs, including SULF1 and SULF2) into FGly, leading to SULF activation [6, 7]. SULF1 was upregulated in UC tumors and promoted cell proliferation in UC cancer cell line and its associated poor overall survival in UC patients [8]. However, the role of the SUMF family in UC has not been elucidated. In this study, we examined genetic alterations and the mRNA and protein expression of SUMFs and related targets in UC.

## Material and methods

### In silico mRNA profile analysis in the bladder of patients with UC

The association between *SUMF2* and the mRNA expression of probable targets was determined using a dataset from The Cancer Genome Atlas (TCGA) (Encyclopedia of RNA Interactomes [ENCORI]; https://starbase.sysu.edu.cn/index.php). Kaplan–Meier analysis (total survival) was performed using the Gene Expression Profiling Interactive Analysis (GEPIA; http://gepia.cancer-pku.cn/) and OncoLnc (http://www.oncolnc.org/) tools on the TCGA dataset.

### Patients and specimen collection

Specimens of bladder tumors were obtained from 192 patients; the bladder cancer tissue array (#BL2081a) was purchased from US Biomax (Rockville, MD, USA). Matched UTUC samples and nearby non-cancerous normal tissues were collected from 155 patients who were surgically treated at the Kaohsiung Veterans General Hospital between 2007 and 2017. The institutional review board of the Kaohsiung Veterans General Hospital approved our study protocol (approval number: VGHKS17-CT4-18). The World Health Organization (WHO) histological standards were used to categorize pathologic grade [9].

### Immunohistochemical (IHC) staining

We performed IHC staining according to the manufacturer’s instructions. In brief, a retrieval solution (Tris EDTA buffer, pH 9.0) was used for 12 min to accomplish antigen retrieval on tissue microarray slides. The slides were then incubated at room temperature for 30 min with a blocking reagent. Next, the slides were incubated with the SUMF2 primary antibody (1:100;11210-1-AP; Proteintech). HistoQuest (version 7.1 Nuclear Segmentation using deep learning, TissueGnostics) was used to assess SUMF2 expression.

### Cell lines and lentivirus infection

5637 human bladder primary cancer cells were acquired from BCRC (#60061). 5637 was cultured in RPMI 1640 with 10% FBS and 1% PSG. From the National RNAi Core Facility (Taipei, Taiwan), lentivirus vector control (pLKO-1-shLuc967) and shSUMF2 shRNA viral supernatant (TRCN0000139923) were acquired. 5367 was infected with 8 μg/ml polybrene using viral supernatants. Using 2 μg/mL puromycin to select cells after 72 h infection.

### Quantitative reverse transcription PCR (RT-qPCR)

TRI Reagent (Sigma-Aldrich, #T9424) was used to extract total RNA, TaKaRa PrimeScript^™^ RT reagent Kit (Cat. #RR037A) was used to synthesize cDNA and PCR reactions were carried out using SYBR system (PCR Biosystems qPCRBIO SyGreen Mix Hi-ROX). The primer sequences were used: *SUMF2*-forward: AGATTTCAGGTACATCAGGGATTT; *SUMF2*-reverse: ACAGACTTC ATTGGCTGGGTG; *ACTB*-forward: AGAAAATCTGGCACCACACC; *ACTB*-reverse: AGAGGCGTACAGGGATAGCA.

### Cell invasion and migration assay

Boyden chambers were used to assess the capacity for cell migration and invasion, as previously mentioned [10]. First, the cells were resuspended in serum-free medium (3 × 10^5^ cells/ml) and loaded into an upper chamber in 50 μl. After 18–24 h, the cells were stained with crystal violet, and the bottom chamber was measured using a light microscope.

### Growth curve assay

A 96-well plate was seeded with 5000 cells/well for 5637/shluc, and 5637/shSUMF2-1. The cells were cultured at 37 °C with 5% CO_2_ for 24–72 h. The 3-(4,5-dimethylthiazol-2-yl)−2,5-diphenyltetrazolium bromide test was used to calculate the cell growth curve.

### Statistical analysis

Based on a median cutoff, tissues expressing SUMF2 were classified as having low or high expression levels. To find out if there were variations in tumor size, tumor grade, and age at sex, chi-square tests were used. The Kaplan–Meier method was used to create survival curves, and the significance of differences between curves was assessed using the log-rank test.

## Results

### *SUMF2* mRNA levels were upregulated and associated with poor clinical outcomes in patients with bladder UC (BLCA)

**Fig. 1:**
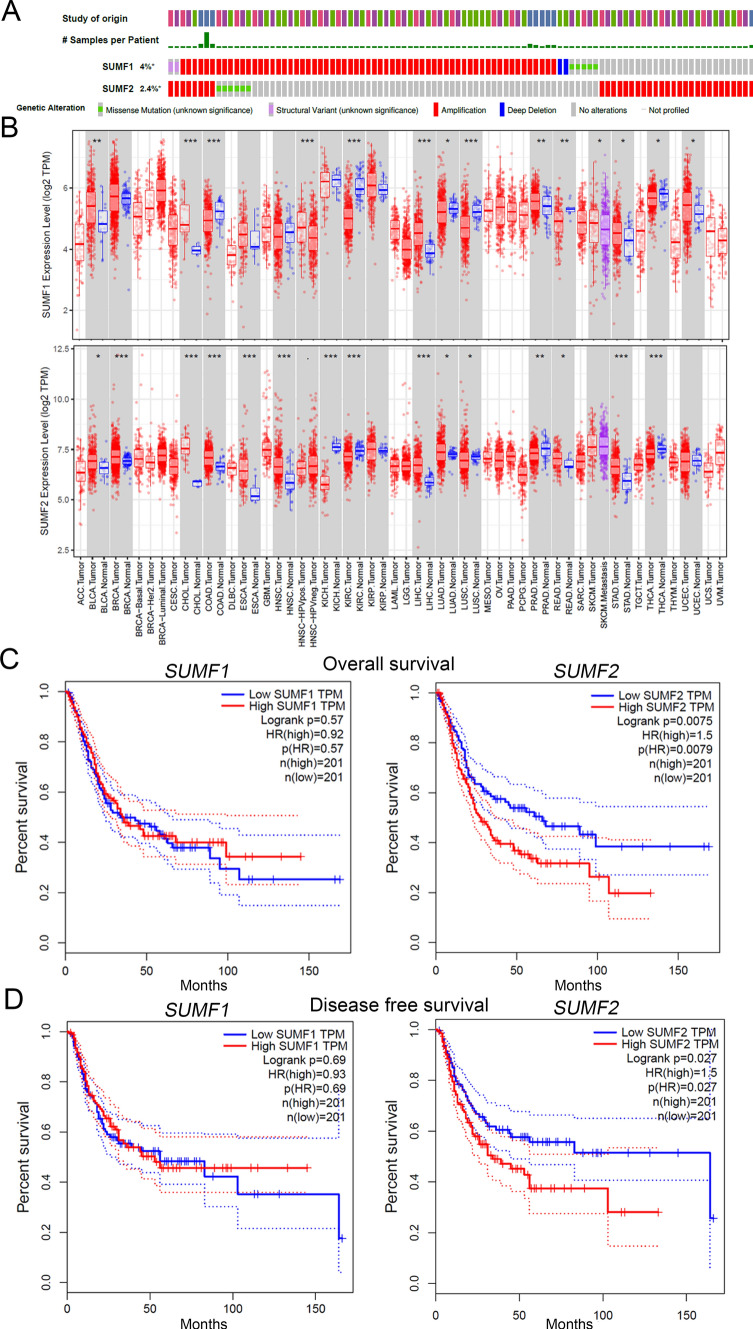
*SUMF2* mRNA expression was associated with poor survival in patients with bladder UC (BLCA). **A** OncoPrint showed *SUMF1* and *SUMF2* amplification in 4% and 2.4% of UC tumors, respectively. Colors indicate the type of genetic change (missense, in-frame, truncated, amplification, deletion, or fusion), and the different cohorts are listed below the OncoPrint. **B** *SUMF1* and *SUMF2* mRNA levels in multiple cancer types. Kaplan–Meier curve for overall survival (**C**) and disease-free survival (**D**), according to the mRNA expression of *SUMF1* and *SUMF2* in patients with BLCA, using TCGA datasets (GEPIA). *HR* hazard ratio

First, we analyzed the genetic profiles of *SUMF1* and *SUMF2* obtained from 4,732 UC samples, which revealed that *SUMF1* and *SUMF2* were significantly amplified in the UC genetic profiles (Fig. 1A). Next, we evaluated the mRNA levels of SUMF family members in patients with multiple cancer types using a publicly available dataset (TIMER). We found that the expression of *SUMF1* and *SUMF2* was upregulated in 8 and 10 different cancer types, respectively (Fig. 1B). Subsequently, to evaluate which members of the *SUMF* family were associated with BLCA progression, we used a TCGA dataset to perform a Kaplan–Meier survival curve analysis (GEPIA). The results showed that *SUMF2* expression levels were associated with poor overall survival (OS) and disease-free survival (DFS) in patients with BLCA (Fig. 1C, D). Therefore, we focused on SUMF2 and proceeded to investigate its clinicopathological characteristics in BLCA using TCGA. We found that high *SUMF2* levels in BLCA tumors were significantly associated with the T status (*P* = 0.023) and lymph node status (*P* = 0.003) (Table 1).

**Table 1:** Correlation of *SUMF2* expression with clinicopathological characteristics in bladder cancer

Variables	Item	Patient No	SUMF2		*P* value
			Low	High	
			No	No	
		372	186	186	
Age (y)	≤ 65	141	72	69	0.831
	> 65	231	114	117	
Sex	Female	97	49	48	1.000
	Male	275	137	138	
T status	1	3	1	2	0.023*
	2	117	70	47	
	3	194	83	111	
	4	58	32	26	
N status	Negative	221	125	96	0.003*
	Positive	151	61	90	
M status	Negative	364	183	181	0.724
	Positive	8	3	5	
Stage	I/II	105	61	44	0.065
	III/IV	267	125	142	

*: A *P*-value of less than 0.05 was deemed statistically significant

### SUMF2 protein levels were upregulated and associated with poor clinical outcomes in patients with UC

**Fig. 2:**
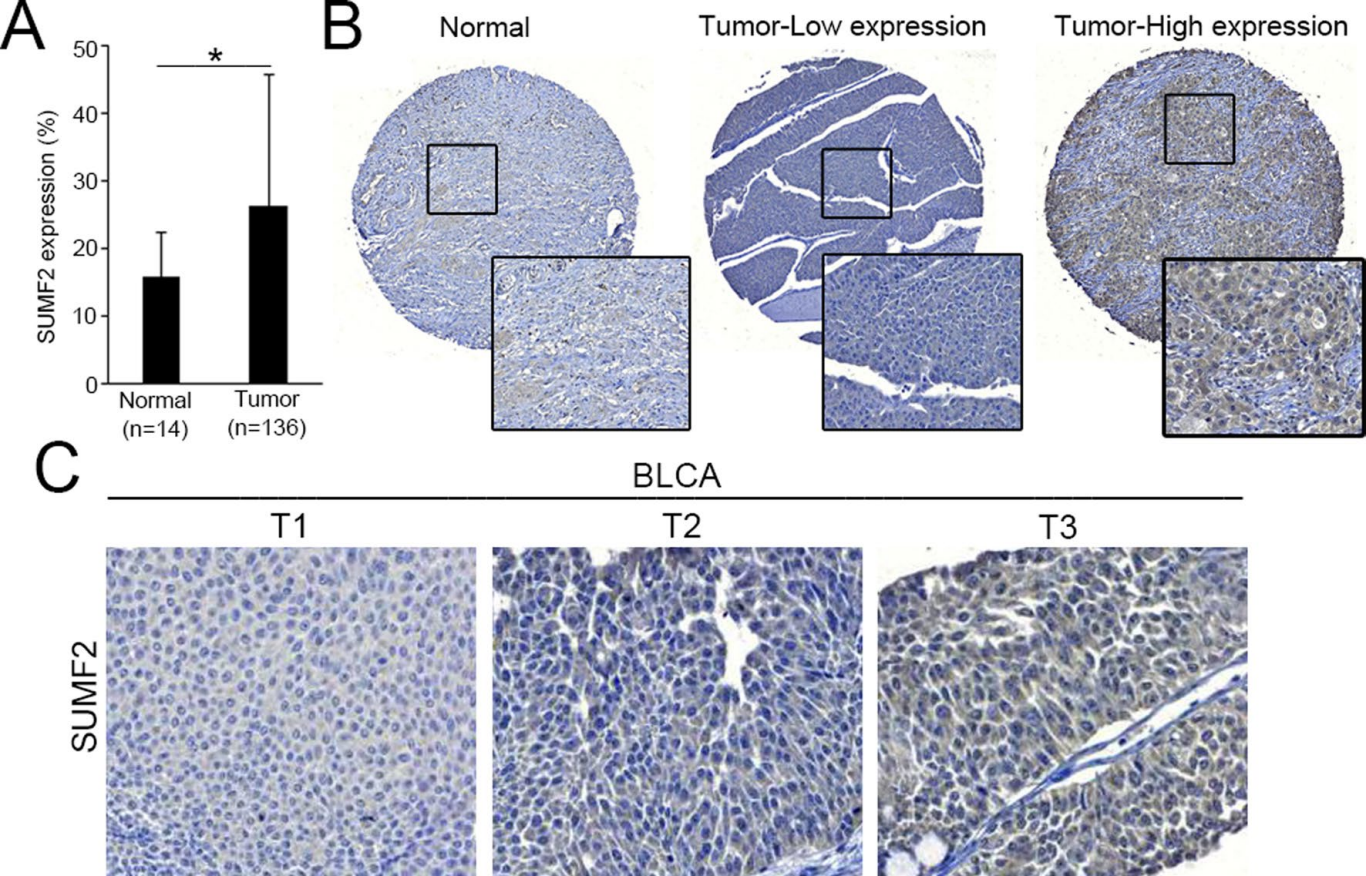
The expression of the SUMF2 protein was elevated in the tissues of bladder cancer. **A** SUMF2 expression in BLCA specimens was quantified, and the Student's *t*-test was used to evaluate significance. *P < 0.05. **B** Representative images of SUMF2 expression in BLCA tissues. **C** SUMF2 expression in different Tumor status of BLCA tissues

**Fig. 3:**
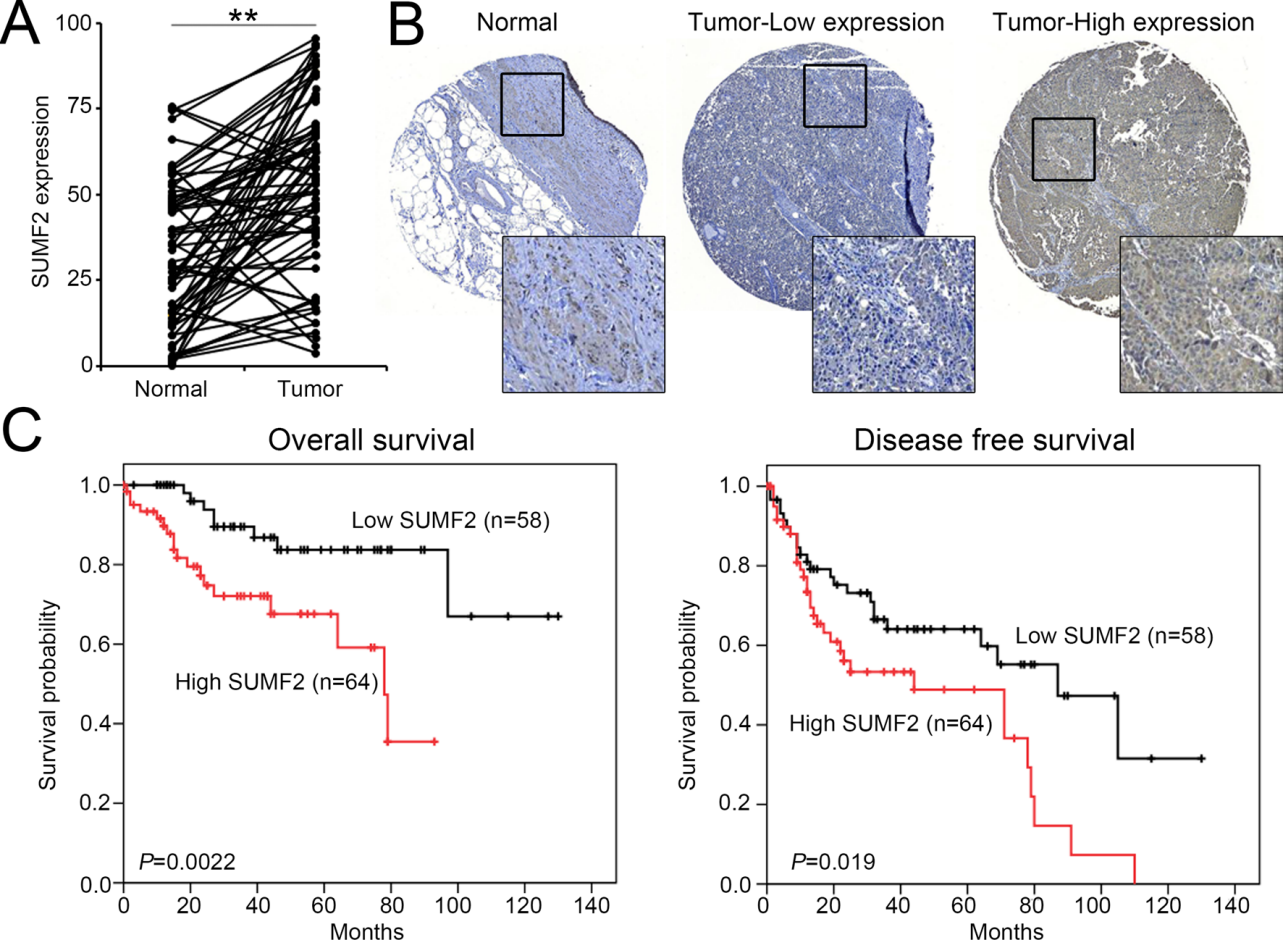
SUMF2 expression was upregulated in UTUC. **A** Quantification of SUMF2 expression in 74 paired UTUC specimens and significance, determined using the Student's *t-*test. **P < 0.01. **B** Representative images of SUMF2 expression in upper tract UC (UTUC) tissues. **C** Kaplan–Meier survival curves for overall survival and disease-free survival in patients with UTUC according to SUMF2 protein expression

To evaluate SUMF2 protein levels in BLCA tissues, we examined the expression of SUMF2 in BLCA tissues using IHC staining. SUMF2 was upregulated in BLCA tumor tissues compared with normal tissues (Fig. 2A). To further evaluate SUMF2 expression in BLCA tumors from 155 patients using IHC and correlated SUMF2 expression with the clinicopathological characteristics of the patients. Based on the levels of SUMF2 protein expression in BLCA tissues, the samples were categorized into low- and high-expression groups, as shown in Fig. 2B. We found that SUMF2 expression levels in BLCA tissues were significantly associated with grade (*P* < 0.001), T status (*P* = 0.011), and stage (*P* = 0.006) (Fig. 2C and Table 2). *SUMF2* mRNA and protein levels were consistently associated with T status. We evaluated further the SUMF2 protein levels in patients with UTUC. Using IHC staining, we analyzed 74 sets of UTUC specimens and paired normal tissues. SUMF2 expression was significantly upregulated in 60 of the 74 samples compared with that in normal tissues (Fig. 3A). Next, we examined SUMF2 expression in UTUC samples from 122 patients using IHC staining and correlated the expression of SUMF2 with the clinicopathological characteristics of these patients. As shown in Fig. 3B, UTUC tissues were classified into two groups, depending on SUMF2 protein expression—low- and high-SUMF2 expression groups. SUMF2 expression levels in UTUC tissues were significantly associated with stage (Table 3, *P* = 0.046). The log-rank test was used to analyze survival rate, and the results showed that the group with high SUMF2 expression levels had lower OS and DFS rates than that with low SUMF2 expression levels (Fig. 3C).

**Table 2:** Correlation of SUMF2 expression with clinicopathological characteristics in bladder cancer

Variables	Item	Patient No	SUMF2		
			Low	High	
			No	No	*P* value
		155	77	78	
Age (y)	≤ 65	100	44	56	0.066
	> 65	55	33	22	
Sex	Female	36	17	19	0.850
	Male	119	60	59	
Grade	Low	107	67	40	< 0.001*
	High	48	10	38	
T status	1	49	29	20	0.011*
	2	83	43	40	
	3	23	5	18	
Stage	I/II	132	72	60	0.006*
	III/IV	23	5	18	

*: A *P*-value of less than 0.05 was deemed statistically significant

**Table 3:** Correlation of SUMF2 expression with clinicopathological characteristics in UTUC

Variables	Item	Patient no	SUMF2			*P* value
			Low		High	
			No		No	
		122	58		64	
Age (y)	≤ 65	48	24		24	0.713
	> 65	74	34		40	
Sex	Female	76	41		35	0.092
	Male	46	17		29	
Grade	Low	2	2		0	0.321
	High	114	53		61	
	–	6	3		3	
T status	1–2	69	38		31	0.069
	3–4	53	22		33	
Stage	I/II	68	38		30	0.046*
	III/IV	54	20		34	

*: A *P*-value of less than 0.05 was deemed statistically significant

### *SUMF2* knockdown reduced migration and invasion abilities of the UC cells

**Fig. 4:**
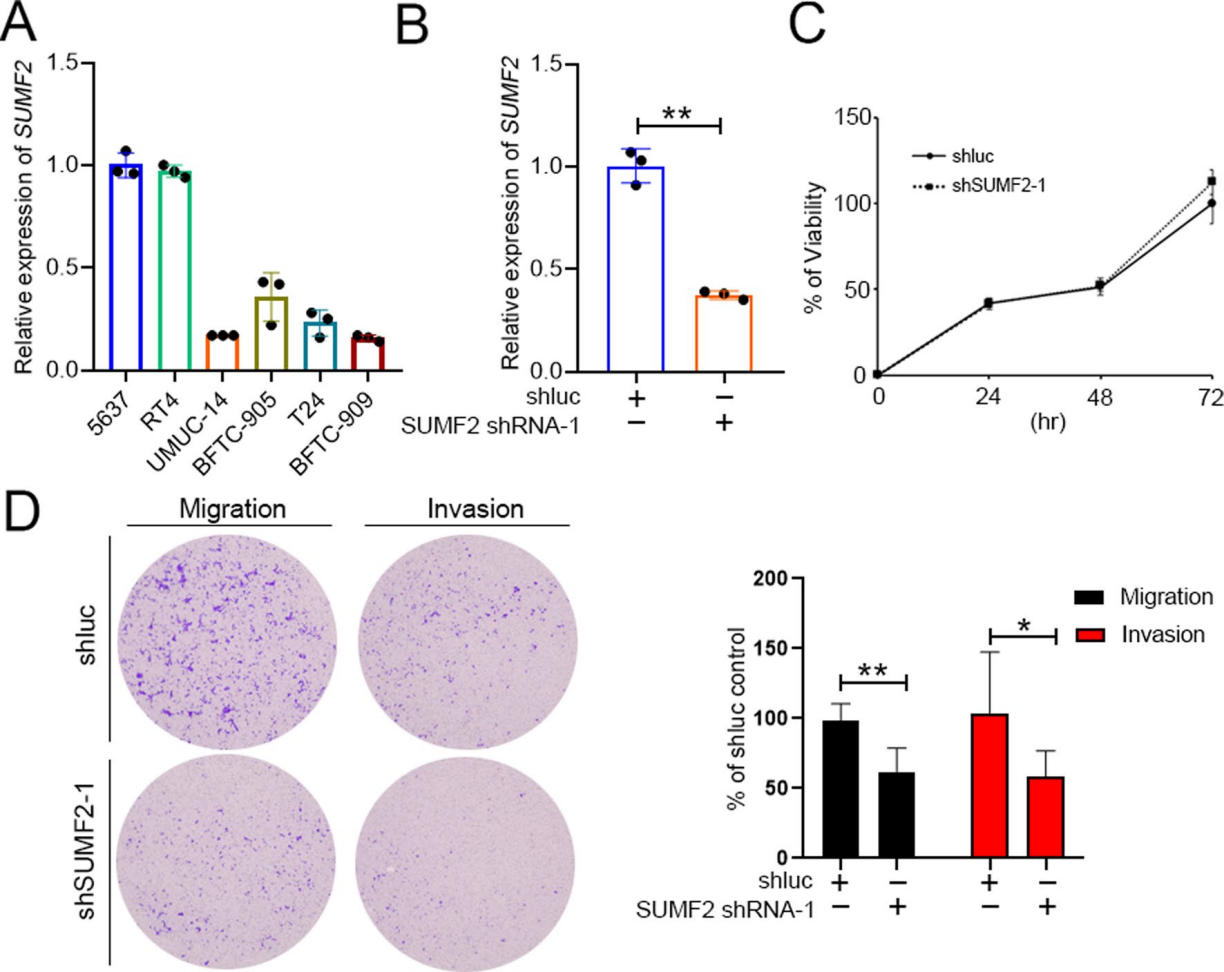
*SUMF2* knockdown inhibits migration and invasion in UC cells. **A** RT-qPCR analysis of *SUMF2* expression in UC cancer cell lines. **B** RT-qPCR examination of *SUMF2* expression following infection with shRNA. **C** The impact of *SUMF2* knockdown on 5637 cell growth. **D** Downregulation of *SUMF2* inhibited migration and invasion viabilities of 5637 cells. Data are presented as mean ± SD. **P < 0.01; *P < 0.05

To evaluate the potential function of SUMF2 in bladder cancer cell lines, we used a BioGRID CRISPR screen summary dataset [11]. SUMF2 function was associated with cell proliferation in UC cell lines (Table S1). To further confirm the effect of SUMF2 on cell proliferation, migration, and invasion in UC cells. We evaluated the expression of *SUMF2* in UC cell lines (Fig. 4A). *SUMF2* was upregulated in 5637 and RT-4 cancer cell lines. Based on the *SUMF2* expression levels, we used 5637 (*SUMF2* high expression) cancer cells to evaluate the effect of SUMF2 on cell proliferation, migration, and invasion. Knockdown of *SUMF2* did not affect the cell viability of 5637 cells (Fig. 4B, C). Moreover, *SUMF2* knockdown significantly reduced the migration and invasion capabilities in 5637 cancer cells (Fig. 4D).

### *SUMF2* levels positively correlated with *SULF1* and* SULF2* in patients with bladder cancer

**Fig. 5:**
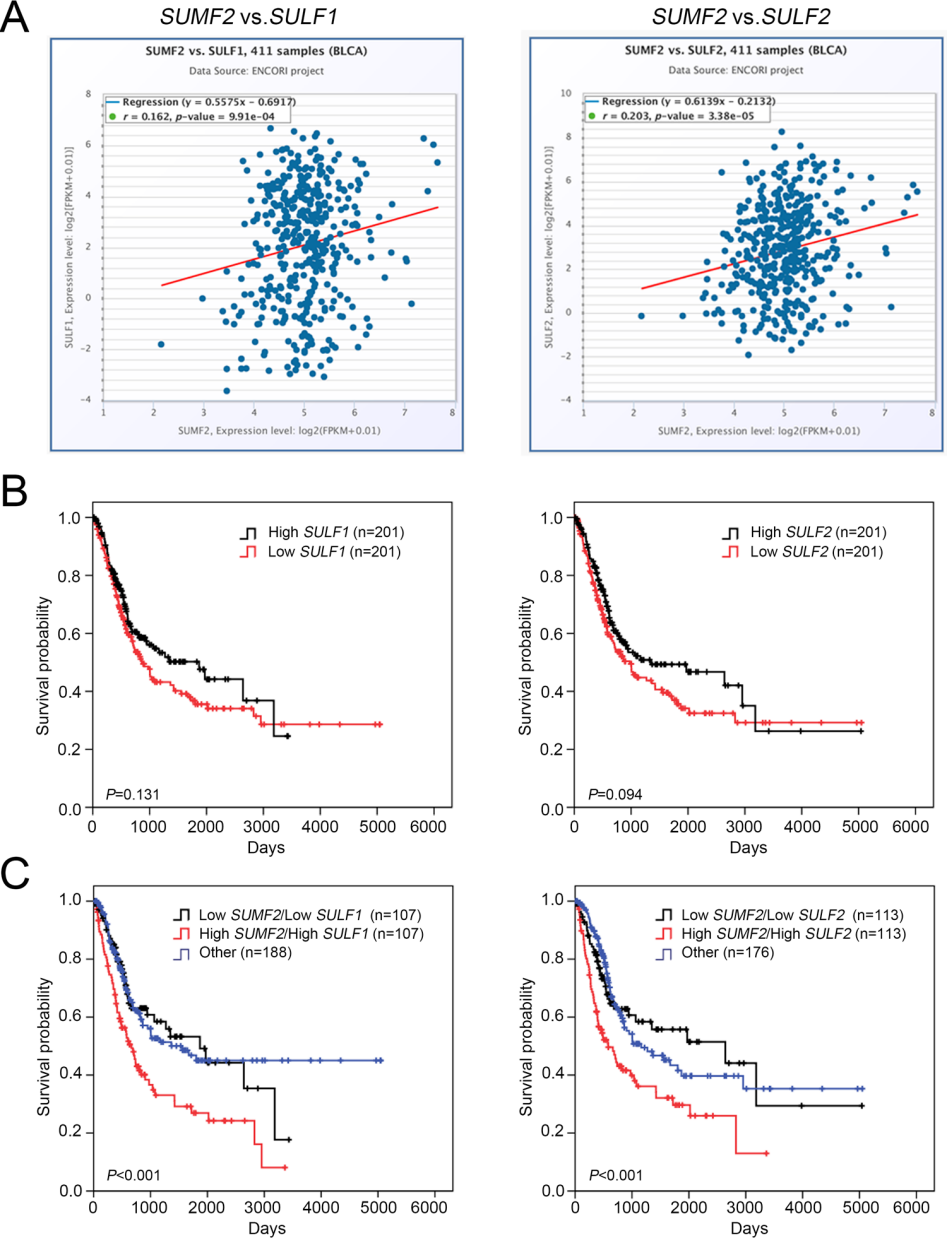
Correlation analysis between *SUMF2* and *SULF* family expression in patients with BLCA. **A** Correlation of *SUMF2* with *SULF1* and *SULF2* mRNA expression using TCGA datasets of BLCA (ENCORI). **B** Kaplan–Meier analysis of overall survival, according to *SULF1* and *SULF2* mRNA expression in BLCA. **C** Kaplan–Meier survival curves for *SUMF2* and *SULF1* and the *SUMF2*/*SULF2* axis in patients with BLCA (using TCGA data)

SUMF is a response to active SULFs via the catalysis of the oxidation of cysteine in the active site of the latter [6, 7]. We confirmed a correlation between the expression of *SUMF2* and the SULF family using TCGA (ENCORI dataset) in patients with BLCA. We found that the *SUMF2* mRNA expression levels significantly correlated with the *SULF1* and *SULF2* expression levels in patients with BLCA (Fig. 5A). Survival analyses showed that *SULF1* and *SULF2* expression was not significantly associated with OS in BLCA (Fig. 5B). Furthermore, among patients with BLCA, the high *SUMF2*/high *SULF1* and high *SUMF2*/high *SULF2* groups were linked to a lower OS than the low *SUMF2*/low *SULF1* and low *SUMF2*/low *SULF2* groups, respectively (Fig. 5C).

### *SUMF2* levels negatively correlated with F-box and WD repeat domain-containing 7 (*FBXW7*) in patients with bladder cancer

**Fig. 6:**
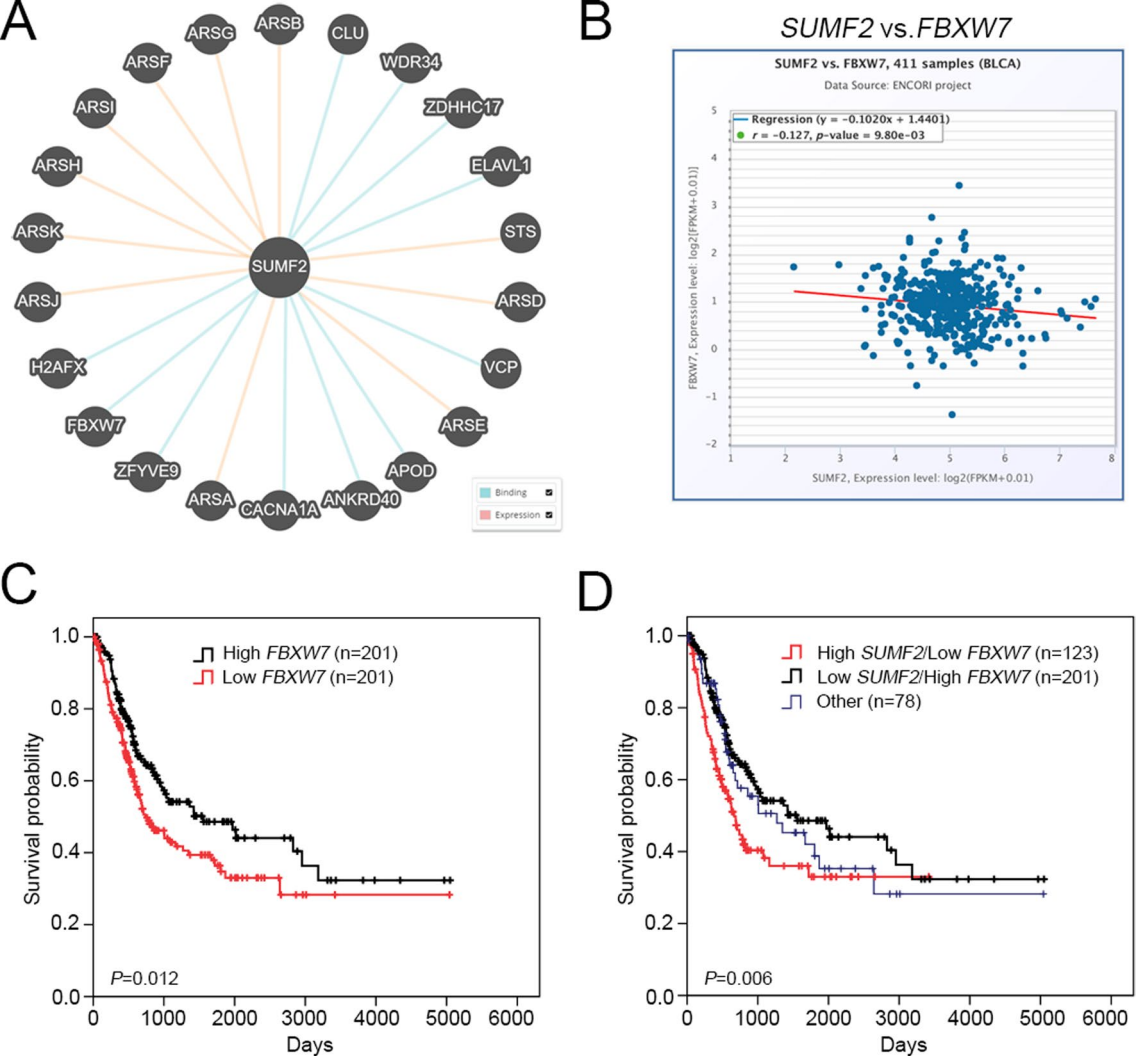
The expression of high *SUMF2*/ low *FBXW7* axis is correlated with poor survival among patients with BLCA. **A** Putative binding and interaction targets of SUMF2 were identified from Pathway Commons. **B** Analysis of the correlation between SUMF2 and *FBXW7* mRNA expression using ENCORI. **C** Analysis of overall survival according to *FBXW7* mRNA expression (TCGA). **D** Kaplan–Meier analysis of overall survival among patients with BLCA by examining the combination of *SUMF2* and *FBXW7* mRNA expression levels. other: SUMF2 low/FBXW7 low and SUMF2 high/FBXW7 high mRNA levels

Based on Pathway Commons database (https://apps.pathwaycommons.org/) and BioGRID dataset (https://thebiogrid.org/), predicted targets were associated with SUMF2, and FBXW7 appeared consistent in both datasets. In addition, the BioGRID dataset, SUMF2 and FBXW7 interaction relationships were derived from the two-hybrid system [12]. Two datasets revealed that SUMF2 binds or interacts with FBXW7 (Fig. 6A and Table S2). We used the ENCORI dataset to evaluate the relationship between *SUMF2* and *FBXW7* in patients with BLCA and other cancer types. We found a significant negative correlation between *SUMF2* and *FBXW7* levels (Fig. 6B and Fig S1). FBXW7 belongs to the F-box protein family and acts as a tumor suppressor against cancer progression [13, 14]. High *FBXW7* levels were linked to longer OS in individuals with bladder cancer (Fig. 6C). Moreover, among patients with bladder cancer, the low *SUMF2*/high *FBXW7* group was associated with longer OS than the high *SUMF2*/low *FBXW7* group (Fig. 6D).

## Discussion

We assessed the expression of SUMF family members in patients with UC and found that *SUMF1* and *SUMF2* were amplified in these patients. The *SUMF2* mRNA expression levels were associated with T status, lymph node metastasis, and worse OS in patients with BLCA. Similar findings regarding SUMF2 protein levels were observed, with high SUMF2 protein levels being associated with high grade, T status, and stage in patients with BLCA. Moreover, high SUMF2 levels were associated with stage and poor OS and DFS in patients with UTUC. Knockdown of *SUMF2* inhibits the migration and invasion abilities of UC cells. *SUMF2* mRNA levels were positively correlated with *SULF1* and *SULF2* mRNA levels and negatively correlated with *FBXW7* levels in patients with BLCA. These findings suggest that SUMF2 may serve as a prognostic marker in UC.

The function of the SUMF family is modulated to activate a SULF site, leading to SULF activation. This mechanism controls the sulfation status of heparan sulfate proteoglycans (HSPGs) [15], which play important roles in many biological processes and organ systems [16]. Upregulated SULF1 expression was associated with T status and high grade in patients with UC. An in vitro model showed that SULF1 overexpression promotes cell proliferation in a UC cell line [8]. Our data showed that *SUMF1* and *SUMF2* levels were upregulated in BLCA tumors, but only *SUMF2* expression levels were significantly associated with worse OS in patients with BLCA. Although *SUMF1* and *SUMF2* expression have similar patterns in BLCA tumors, SUMF2 lacks the enzymatic activity necessary for the production of FGly due to the absence of two catalytic cysteines (Cys336 and Cys341) compared with the SUMF1 active site [17]. In addition, SUMF2 can bind to SUMF1 and SULF and control the actions of these enzymes as well as the FGly forming process [7]. We also observed that the *SUMF2* levels positively correlated with the *SULF1* and *SULF2* levels in patients with BLCA. OS significantly decreased in the high *SUMF2*/high *SULF1* and high *SUMF2*/high *SULF2* expression cohorts compared with that in the low *SUMF2*/low *SULF1* and low *SUMF2*/low *SULF2* group, respectively. These findings suggest that the *SUMF2*/*SULF1* and *SUMF2*/*SULF2* axes may play a role in UC progression.

FBXW7 is a substrate receptor of the SCF E3 ubiquitin ligase and a tumor suppressor [13, 18, 19], which can enhance the ubiquitination and degradation of oncogene proteins, resulting in inhibition of cancer cell growth, including of UC cells [20]. In our study, we observed SUMF2 interaction with the tumor suppressor FBXW7 using a BioGRID dataset and Pathway Commons analysis. *SUMF2* levels negatively correlated with the expression of *FBXW7* in patients with BLCA. The high *SUMF2*/low *FBXW7* expression BLCA cohort showed poor OS compared with that in the low *SUMF2*/ high *FBXW7* cohort. We could not exclude the possibility that oncogenic proteins interact with FBXW7 leading to reduced OS in UC patients; however, this is the first study to investigate that *SUMF2/FBWX7* axis associated with poor OS in UC patients, providing the basis for prognostic marker of UC.

## Conclusions

This study is the first to assess the expression of genetic variants of the SUMF family in patients with UC. Compared to low SUMF2 expression levels, high SUMF2 expression levels were associated with shorter OS, suggesting that SUMF2 expression may act as an independent predictor of UC outcomes.

## Supplementary Information

Supplementary material 1

## Data Availability

Data Availability The datasets used and analyzed during the current study are available from the corresponding author upon reasonable request.

## Abbreviations

BLCA: Bladder urothelial carcinoma
DFS: Disease-free survival
FBXW7: F-box and WD repeat domain containing 7
FGly: Cα-formylglycine
FGE: Formylglycine-generating enzyme
HSPGs: Heparan sulfate proteoglycans
IHC: Immunohistochemistry
SUMF1: Sulfatase modifying factor 1
SUMF2: Sulfatase modifying factor 2
OS: Overall survival
SULFs: Sulfatases
SULF1: Sulfatase-1
SULF2: Sulfatase-2
TCGA: The Cancer Genome Atlas
UTUC: Upper tract urothelial carcinoma
UC: Urothelial carcinoma
BRCA: Breast invasive carcinoma
COAD: Colon adenocarcinoma
KIRC: Kidney renal clear cell carcinoma
LAML: Acute Myeloid Leukemia
LUAD: Lung adenocarcinoma
LUSC: Lung squamous cell carcinoma
