# Supplementary material for: Sulfatase modifying factor 2 as a predictive biomarker for urothelial carcinoma
Source: Discov Oncol. 2025 Feb 7;16:126. doi: 10.1007/s12672-025-01859-y (PMC11803025; doi:10.1007/s12672-025-01859-y)
Supplement: Supplementary file 1 — Supplementary material 1 [file 12672_2025_1859_MOESM1_ESM.docx]

**Sulfatase modifying factor 2 as a predictive biomarker for urothelial carcinoma**

**Wei-Ting Kuo^1,2^, Yi-Chen Lee^3^, Jia-Bin Liao^4^, Ching-Jiunn Tseng^2,5^, and Yi-Fang Yang^5^***

https://orcid.org/0000-0002-4889-095X (Yi-Chen Lee)

https://orcid.org/0000-0001-7425-3156 (Yi-Fang Yang)

^1^Division of Urology, Department of Surgery, Kaohsiung Veterans General Hospital, Kaohsiung, Taiwan

^2^Institute of Clinical Medicine, National Yang Ming Chiao Tung University, Taiwan

^3^Department of Anatomy, School of Medicine, College of Medicine, Kaohsiung Medical University, Kaohsiung, Taiwan

^4^Department of Pathology and Laboratory Medicine, Kaohsiung Veterans General Hospital, Kaohsiung, Taiwan

^5^Department of Medical Education and Research, Kaohsiung Veterans General Hospital, Kaohsiung, Taiwan

**Address requests for reprints/correspondence to:**

**Yi-Fang Yang**, Department of Medical Education and Research, Kaohsiung Veterans General Hospital, No. 386, Dajhong 1st Rd., Zuoying Dist., Kaohsiung City 813414, Taiwan. Phone: 886-7-342-2121 #71592; Fax: 886-7-342-2288; E-mail: yvonne845040@gmail.com

**Supplementary information**

**Figure S1 Related Figure 6**

**Table S1 Related Figure 4**

**Table S2 Related Figure 6**

**Figure S1**


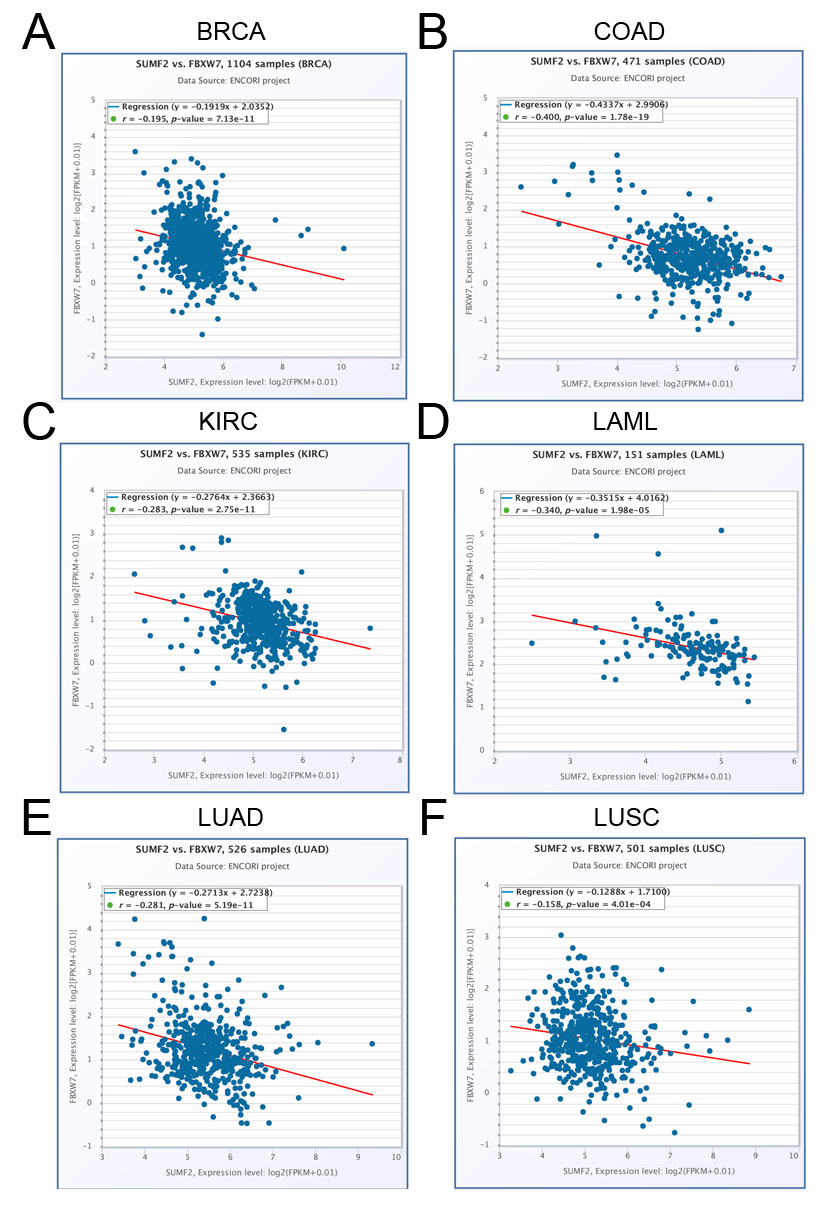


Figure S1. *SUMF2* and *FBXW7* were significantly negatively correlated in patients with BRCA, COAD, KIRS, LAML, LUAD, and LUSC.BRCA: Breast invasive carcinoma; COAD: Colon adenocarcinoma; KIRC: Kidney renal clear cell carcinoma; LAML: Acute Myeloid Leukemia; LUAD: Lung adenocarcinoma; LUSC: Lung squamous cell carcinoma.

Table S1. CRISPR Screen Summary of SUMF2 in bladder cancer cell lines.

| SCREEN_NAME | LIBRARY  _TYPE | LIBRARY  _METHODOLOGY | CELL_LINE | CELL_TYPE | PHENOTYPE | ORGANISM  _OFFICIAL | SOURCE | SCREEN_RATIONALE |
| --- | --- | --- | --- | --- | --- | --- | --- | --- |
| PMID29083409 | CRISPRn | Knockout | VM-CUB-1 | Bladder Carcinoma | cell proliferation | H. sapiens | BioGRID ORCS | Cell-essential genes |
| PMID29083409 | CRISPRn | Knockout | JMSU-1 | Bladder Carcinoma | cell proliferation | H. sapiens | BioGRID ORCS | Cell-essential genes |
| PMID29083409 | CRISPRn | Knockout | KU-19-19 | Bladder Carcinoma | cell proliferation | H. sapiens | BioGRID ORCS | Cell-essential genes |
| PMID29083409 | CRISPRn | Knockout | RT112/84 | Bladder Carcinoma | cell proliferation | H. sapiens | BioGRID ORCS | Cell-essential genes |
| PMID29083409 | CRISPRn | Knockout | SLR20 | Bladder Carcinoma | cell proliferation | H. sapiens | BioGRID ORCS | Cell-essential genes |
| PMID29083409 | CRISPRn | Knockout | TCCSUP | Bladder Carcinoma | cell proliferation | H. sapiens | BioGRID ORCS | Cell-essential genes |
| PMID29083409 | CRISPRn | Knockout | CAL-29 | Bladder Carcinoma | cell proliferation | H. sapiens | BioGRID ORCS | Cell-essential genes |
| PMID36597481 | CRISPRn | Knockout | UM-UC-4 | Bladder Carcinoma | cell proliferation | H. sapiens | BioGRID ORCS | Cell-essential genes |
| PMID29083409 | CRISPRn | Knockout | UM-UC-3 | Bladder Transitional Cell Carcinoma Cell Line | cell proliferation | H. sapiens | BioGRID ORCS | Cell-essential genes |
| PMID29083409 | CRISPRn | Knockout | RT-4 | Bladder Transitional Cell Carcinoma Cell Line | cell proliferation | H. sapiens | BioGRID ORCS | Cell-essential genes |
| PMID29083409 | CRISPRn | Knockout | RT-112 | Bladder Transitional Cell Carcinoma Cell Line | cell proliferation | H. sapiens | BioGRID ORCS | Cell-essential genes |

Table S2. Interactors protein of SUMF2.

| #BioGRID Interaction ID | Official Symbol Interactor A | Official Symbol Interactor B | Experimental System | Organism Name Interactor A | Organism Name Interactor B |
| --- | --- | --- | --- | --- | --- |
| 2625957 | AK1 | SUMF2 | Co-fractionation | Homo sapiens | Homo sapiens |
| 2624866 | AK2 | SUMF2 | Co-fractionation | Homo sapiens | Homo sapiens |
| 2624750 | AK4 | SUMF2 | Co-fractionation | Homo sapiens | Homo sapiens |
| 2633774 | APEX1 | SUMF2 | Affinity Capture-RNA | Homo sapiens | Homo sapiens |
| 3096862 | ARSK | SUMF2 | Affinity Capture-MS | Homo sapiens | Homo sapiens |
| 3645343 | B3GNT2 | SUMF2 | Affinity Capture-MS | Homo sapiens | Homo sapiens |
| 2874915 | BCAR1 | SUMF2 | Affinity Capture-MS | Homo sapiens | Homo sapiens |
| 570011 | CACNA1A | SUMF2 | Two-hybrid | Homo sapiens | Homo sapiens |
| 2981506 | CALR3 | SUMF2 | Proximity Label-MS | Homo sapiens | Homo sapiens |
| 2981589 | CALU | SUMF2 | Proximity Label-MS | Homo sapiens | Homo sapiens |
| 3349671 | CANX | SUMF2 | Affinity Capture-MS | Homo sapiens | Homo sapiens |
| 3501671 | CDK7 | SUMF2 | Affinity Capture-MS | Homo sapiens | Homo sapiens |
| 3437774 | CKMT1B | SUMF2 | Co-fractionation | Homo sapiens | Homo sapiens |
| 2628511 | DGUOK | SUMF2 | Co-fractionation | Homo sapiens | Homo sapiens |
| 3410391 | E2F4 | SUMF2 | Affinity Capture-MS | Homo sapiens | Homo sapiens |
| 664347 | ELAVL1 | SUMF2 | Affinity Capture-RNA | Homo sapiens | Homo sapiens |
| 838502 | env | SUMF2 | Affinity Capture-MS | Human Immunodeficiency Virus 1 | Homo sapiens |
| 2628631 | FAHD1 | SUMF2 | Co-fractionation | Homo sapiens | Homo sapiens |
| 558198 | H2AFX | SUMF2 | Affinity Capture-MS | Homo sapiens | Homo sapiens |
| 2655345 | LMBR1L | SUMF2 | Affinity Capture-MS | Homo sapiens | Homo sapiens |
| 2995129 | LRRC59 | SUMF2 | Proximity Label-MS | Homo sapiens | Homo sapiens |
| 2629149 | LYPLA1 | SUMF2 | Co-fractionation | Homo sapiens | Homo sapiens |
| 2624935 | LYPLAL1 | SUMF2 | Co-fractionation | Homo sapiens | Homo sapiens |
| 2876159 | nsp4ab | SUMF2 | Affinity Capture-MS | Middle-East Respiratory Syndrome-related Coronavirus | Homo sapiens |
| 3546031 | nsp5 | SUMF2 | Biochemical Activity | Severe acute respiratory syndrome coronavirus 2 | Homo sapiens |
| 3580696 | ORF10 | SUMF2 | Proximity Label-MS | Severe acute respiratory syndrome coronavirus 2 | Homo sapiens |
| 2755948 | ORF3a | SUMF2 | Affinity Capture-MS | Severe acute respiratory syndrome coronavirus 2 | Homo sapiens |
| 2906927 | ORF3a | SUMF2 | Affinity Capture-MS | Severe acute respiratory syndrome coronavirus 2 | Homo sapiens |
| 3501414 | ORF3a | SUMF2 | Two-hybrid | Severe acute respiratory syndrome coronavirus 2 | Homo sapiens |
| 2797066 | ORF8 | SUMF2 | Proximity Label-MS | Severe acute respiratory syndrome coronavirus 2 | Homo sapiens |
| 3580418 | ORF8 | SUMF2 | Proximity Label-MS | Severe acute respiratory syndrome coronavirus 2 | Homo sapiens |
| 2999414 | PDIA4 | SUMF2 | Proximity Label-MS | Homo sapiens | Homo sapiens |
| 3396897 | PLD4 | SUMF2 | Affinity Capture-MS | Homo sapiens | Homo sapiens |
| 3005982 | SEC61B | SUMF2 | Proximity Label-MS | Homo sapiens | Homo sapiens |
| 2626395 | SOD1 | SUMF2 | Co-fractionation | Homo sapiens | Homo sapiens |
| **270000** | **SUMF2** | **FBXW7** | **Two-hybrid** | **Homo sapiens** | **Homo sapiens** |
| 1182072 | SUMF2 | ANKRD40 | Affinity Capture-MS | Homo sapiens | Homo sapiens |
| 1182073 | SUMF2 | APOD | Affinity Capture-MS | Homo sapiens | Homo sapiens |
| 1182074 | SUMF2 | CLU | Affinity Capture-MS | Homo sapiens | Homo sapiens |
| 1182075 | SUMF2 | WDR34 | Affinity Capture-MS | Homo sapiens | Homo sapiens |
| 2241831 | SUMF2 | APOD | Affinity Capture-MS | Homo sapiens | Homo sapiens |
| 2248515 | SUMF2 | ANKRD40 | Affinity Capture-MS | Homo sapiens | Homo sapiens |
| 2625943 | SUMF2 | GRHPR | Co-fractionation | Homo sapiens | Homo sapiens |
| 2626657 | SUMF2 | ABHD11 | Co-fractionation | Homo sapiens | Homo sapiens |
| 2627200 | SUMF2 | MSRB2 | Co-fractionation | Homo sapiens | Homo sapiens |
| 2630174 | SUMF2 | AK3 | Co-fractionation | Homo sapiens | Homo sapiens |
| 2630175 | SUMF2 | COA4 | Co-fractionation | Homo sapiens | Homo sapiens |
| 2630176 | SUMF2 | TXN2 | Co-fractionation | Homo sapiens | Homo sapiens |
| 3117328 | SUMF2 | APOD | Affinity Capture-MS | Homo sapiens | Homo sapiens |
| 3130449 | SUMF2 | ANKRD40 | Affinity Capture-MS | Homo sapiens | Homo sapiens |
| 3151249 | SUMF2 | CCT3 | Affinity Capture-MS | Homo sapiens | Homo sapiens |
| 3152791 | SUMF2 | HSPA2 | Affinity Capture-MS | Homo sapiens | Homo sapiens |
| 3179161 | SUMF2 | TUBA1A | Affinity Capture-MS | Homo sapiens | Homo sapiens |
| 3182851 | SUMF2 | CCT6B | Affinity Capture-MS | Homo sapiens | Homo sapiens |
| 3452791 | SUMF2 | FUBP3 | Co-fractionation | Homo sapiens | Homo sapiens |
| 3008736 | SYNE3 | SUMF2 | Proximity Label-MS | Homo sapiens | Homo sapiens |
| 3563907 | TGOLN2 | SUMF2 | Proximity Label-MS | Homo sapiens | Homo sapiens |
| 3306207 | TMEM106B | SUMF2 | Proximity Label-MS | Homo sapiens | Homo sapiens |
| 2630295 | TRIAP1 | SUMF2 | Co-fractionation | Homo sapiens | Homo sapiens |
| 3522333 | TRIM67 | SUMF2 | Affinity Capture-MS | Homo sapiens | Homo sapiens |
| 2627572 | TXNDC12 | SUMF2 | Co-fractionation | Homo sapiens | Homo sapiens |
| 2534217 | VCP | SUMF2 | Affinity Capture-MS | Homo sapiens | Homo sapiens |
| 1056291 | ZDHHC17 | SUMF2 | Two-hybrid | Homo sapiens | Homo sapiens |
| 834010 | ZFYVE9 | SUMF2 | Two-hybrid | Homo sapiens | Homo sapiens |
| 3471284 | ZRANB1 | SUMF2 | Affinity Capture-MS | Homo sapiens | Homo sapiens |
